# Coronavirus vaccination coverage and timeliness among people experiencing homelessness in Wales, UK: A population-level analysis of linked healthcare and substance use services data.

**DOI:** 10.23889/ijpds.v7i3.1884

**Published:** 2022-08-25

**Authors:** Ian Thomas

**Affiliations:** 1 Administrative Data Research Wales

## Objectives

People experiencing homelessness (PEH) have higher incidence of underlying health conditions that increase the risk of COVID-19 related complications. Achieving high vaccination coverage in a timely manner was therefore important for this group. We aimed to quantify the coverage and the timeliness of coronavirus vaccination among PEH in Wales.

## Approach

Analysis was conducted using a population spine of people ≥ 18 years old continuously resident in Wales, UK, between July 2020 and November 2021. Data from health and substance use services were used to flag people who had experienced homelessness. The percentage of people receiving at least one dose of the coronavirus vaccination between 2nd December 2020 and 30th November 2021 was derived and logistic regression used to estimate the effect of experiencing homelessness on the odds of vaccination. Cumulative incidence plots and Restricted Meant Survival Time analysis were used to examine the timeliness of vaccination by homelessness status.

## Results

The proportion of people vaccinated by 30th November 2021 was roughly 26% points lower among PEH than ‘not-homeless’ people. Adjusted logistic regression found that people experiencing homelessness had lower odds of having received a first vaccine dose by 30th November 2021 (OR = 0.4, p < 0.01). Since the start of the mass vaccination programme in Wales in December 2020, PEH went unvaccinated for on average 90 days longer than the general ‘not-homeless’ population, and 55 days longer than an exact match ‘not-homeless’ group. Visual analysis of the cumulative incidence plots found that at all points during the mass vaccination programme in Wales, the probability of being vaccinated was lower for PEH than ‘not-homeless’ groups.

## Conclusion

PEH appear to have been under-engaged as part of the mass vaccination programme in Wales. This has led to lower overall vaccination rates and more time unvaccinated, potentially exposing PEH to greater risk of COVID-19 complications and mortality.

